# The role of sleep on cognition and functional connectivity in patients with multiple sclerosis

**DOI:** 10.1007/s00415-016-8318-6

**Published:** 2016-10-24

**Authors:** Quinten van Geest, B. Westerik, Y. D. van der Werf, J. J. G. Geurts, H. E. Hulst

**Affiliations:** Department of Anatomy and Neurosciences, VU University Medical Center, Amsterdam Neuroscience, VUmc MS Center Amsterdam, De Boelelaan 1108, room 13 W01, 1081 HZ Amsterdam, The Netherlands

**Keywords:** Multiple sclerosis, Cognition, Sleep, Functional connectivity, fMRI

## Abstract

Sleep disturbances are common in multiple sclerosis (MS), but its impact on cognition and functional connectivity (FC) of the hippocampus and thalamus is unknown. Therefore, we investigated the relationship between sleep disturbances, cognitive functioning and resting-state (RS) FC of the hippocampus and thalamus in MS. 71 MS patients and 40 healthy controls underwent neuropsychological testing and filled out self-report questionnaires (anxiety, depression, fatigue, and subjective cognitive problems). Sleep disturbances were assed with the five-item version of the Athens Insomnia Scale. Hippocampal and thalamic volume and RS FC of these regions were determined. Twenty-three patients were categorized as sleep disturbed and 48 as normal sleeping. No differences were found between disturbed and normal sleeping patients concerning cognition and structural MRI. Sleep disturbed patients reported more subjective cognitive problems, and displayed decreased FC between the thalamus and middle and superior frontal gyrus, inferior frontal operculum, anterior cingulate cortex, inferior parietal gyrus, precuneus, and angular gyrus compared to normal sleeping patients. We conclude that sleep disturbances in MS are not (directly) related to objective cognitive functioning, but rather to subjective cognitive problems. In addition, sleep disturbances in MS seem to coincide with a specific pattern of decreased thalamic FC.

**Electronic supplementary material:**

The online version of this article (doi:10.1007/s00415-016-8318-6) contains supplementary material, which is available to authorized users.

## Introduction

Up to 65 % of multiple sclerosis (MS) patients suffer from cognitive problems [1], resulting in a reduced quality of life [2]. Several factors are thought to negatively influence cognition in MS patients, such as depression [3], fatigue [4], and sleep disturbances [5]. Approximately 50 % of the patients with MS suffer from sleep disturbances (e.g., insomnia or sleep-disordered breathing) [6].

In healthy controls (HCs), proper sleep is important for memory consolidation [7] and sleep deprivation has been related to impaired functioning in various cognitive domains [8]. The literature on sleep disturbances and cognition in MS is scarce. One study showed an association between sleep disturbances and a decline in sustained attention [9], whereas another study related reduced sleep efficiency to problems with information processing and executive function [5].

On functional (f) magnetic resonance imaging (MRI), the effects of sleep disturbances can be seen as hypo-activation in medial and inferior prefrontal areas in subjects with insomnia compared to HCs during a cognitive task, which returned to normal values after sleep therapy [10]. In addition, shallow sleep has been related to reduced hippocampal activation [11], and the thalamus showed decreased functional connectivity (FC) in sleep deprived HCs [12].

In MS, damage to the hippocampus and thalamus (e.g., lesions and atrophy) is associated with worse cognition [13, 14]. In HCs, both regions can be related to sleep and cognition. In the present study, we investigated sleep disturbances in MS in relation to cognitive functioning and resting-state (RS) FC of the hippocampus and thalamus. We hypothesize that sleep problems negatively influence cognition and can be related to FC alterations of the hippocampus and thalamus.

## Materials and methods

### Participants

All patients (*n* = 71; 47 female; mean disease duration 11.0 years) were diagnosed with clinically definite MS according to the revised McDonald criteria [15]. On the day of scanning, disease severity was measured using a questionnaire based on the expanded disability status scale [16]. Age- and sex matched HCs (*n* = 40; 26 female) were included. Subjects included in this study are partly overlapping with a previously reported fMRI study [17]. Exclusion criteria were the presence or history of psychiatric or neurological diseases (for patients: other than MS) and contra-indications for MRI. All participants gave written informed consent prior to participation. The institutional ethical review board approved the study protocol and it has therefore been performed in accordance with the ethical standards laid down in the 1964 Declaration of Helsinki.

### Sleep disturbances

The Athens Insomnia Scale (AIS) is a self-report questionnaire, validated in HCs, and was used to measure sleep disturbances [18]. This questionnaire includes eight items on which a score ranging from zero to three points (no to severe problems) can be obtained for each item. As the eight-item version of the AIS includes three items that can reflect MS symptoms independent from sleep problems (e.g., fatigue during the day), the five-item version of the AIS was used. This version assesses difficulty with sleep quality and quantity, and includes the following items: sleep induction time, awakening during the night, final awakening earlier than desired, total sleep duration, and overall quality of sleep. We categorized patients as ‘sleep disturbed’ if they scored at least three points (which is the median score of patients) with a prerequisite that at least one item should be scored ≥2 (moderate to severe problems). Otherwise, patients were categorized as ‘normal sleeping’.

### Neuropsychological evaluation

All subjects underwent an extensive neuropsychological test battery, consisting of the following tests:

- The Dutch equivalent of the California Verbal Learning Test, the Verbale Leer- en Geheugen Taak (VLGT) [19], to assess verbal learning and memory;
- Letter Digit Substitution Task (LDST; an adaptation of the symbol digit modalities test) [20], to assess information processing speed;
- Location Learning Test (LLT) [21], to assess visuospatial memory;
- Digit Span forward and backward, subtests of the Wechsler Adult Intelligence Scale [22], to assess short term and working memory, respectively;
- World List Generation, including three categories: animals, professions, and m-words (1 min per subtest), to asses verbal fluency [23].

All test scores were converted into *Z*-scores relative to HCs. For each subject, all *Z*-scores were averaged to obtain an average cognition score. Patients were categorized as cognitively impaired if they scored at least two standard deviations below that of HCs on at least two out of five tests. Otherwise, patients were classified as cognitively preserved.

Symptoms of depression, anxiety, fatigue, and subjective cognitive problems were assessed using the Hospital Anxiety and Depression Scale (HADS) [24], the Checklist of Individual Strength (CIS-20) [25], and the Cognitive Function Scale (CFS) for subjective cognitive functioning from the Medical Outcomes study [26].

### MRI acquisition

All subjects were scanned on 1.5T (Siemens Sonata, Erlangen, Germany). Structural MRI consisted of 3DT1-weighted magnetization prepared rapid acquisition gradient-echo (MPRAGE) images and turbo spin-echo proton density (PD)/T2-weighted images. RS fMRI was performed to calculate FC.

### Structural MRI analysis

All imaging processing steps were performed in FSL 5.0 (FMRIB’s Software Library, http://www.fmrib.ox.ac.uk/fsl). Gray matter (GM) and white matter (WM) volumes were obtained using the MPRAGE images and SienaX [27]. FIRST [28] was used to measure the volume of the hippocampus and thalamus. All volumetric measures were normalized for head size. White matter lesions were manually marked and outlined on the PD/T2-weighted scan using a local threshold technique.

### Functional MRI analysis

See the online resources for a detailed description of the FC analysis. In brief, the Automated Anatomical Labelling (AAL) atlas [29] was registered to each subject’s fMRI scan in native space to which subcortical structures were added. This novel atlas, containing 92 regions, was masked for GM, and from each atlas region, the average time series was obtained. FC was calculated between the hippocampus and thalamus (bilateral) and all other brain areas using synchronization likelihood (SL) [30] in BrainWave (http://home.kpn.nl/stam7883/index.html). SL is a measure for linear and nonlinear correlations and ranges from zero to one, and has been previously applied in MS [14].

### Statistical analysis

All analyses were performed using the Statistical Package for the Social Sciences (SPSS, Chicago, IL) version 20. All statistical analyses were performed on group level. Normality of the data was tested with the Kolmogorov–Smirnov test. T2 lesion load was log-transformed, and SL values were inverted (1/SL) to achieve normality; all the other non-normally distributed variables were tested with the Mann–Whitney *U* test. General linear models were used to assess group differences. Univariate and multivariate regression analyses were used to predict AIS score and overall cognitive functioning in MS. A *p* value of 0.05 was considered statistically significant for demographic, behavioural, and structural MRI data and for the regression analyses. To be more conservative concerning the multiple comparisons in the FC analysis, a significance level of 0.01 was used.

Correlations between volumes of the left and right hippocampus and left and right thalamus were 0.73 and 0.95, respectively. Therefore, volumes of the left and right hippocampus and left and right thalamus were added up and treated as single measures to limit the number of variables in the analyses. In the FC analysis, the left and right hippocampus and thalamus were analysed separately, to be more specific.

## Results

### Demographics, neuropsychological evaluation, and MRI in MS vs. HCs

See Supplementary Table 1 for detailed information concerning the demographics and cognitive test scores of patients and HCs. Patients and HCs did not differ significantly with respect to age (mean age patients 45.7 years; mean age HCs 44.0 years; *p* = 0.338), sex (*p* = 0.898), and educational level (median educational level HCs and patients: 6.00; *p* = 0.627). Patients show higher levels of anxiety (*p* = 0.001), depression (*p* < 0.001), fatigue (*p* < 0.001), subjective cognitive problems (*p* < 0.001), and sleep disturbances (*p* = 0.002) compared to HCs. No significant relationship was found between AIS score, fatigue, and subjective cognitive problems in all MS patients. In HCs, higher AIS score was positively correlated with fatigue (Spearman’s *ρ* = 0.57, *p* < 0.001).

Patients performed worse on all cognitive tests compared to HCs. In MS, no significant relationship was found between AIS score and overall objective cognitive functioning or individual neuropsychological test scores. However, in HCs, higher AIS score was correlated with worse LDST performance (Spearman’s *ρ* = −0.35, *p* = 0.026).

MS patients had reduced normalized GM volume (NGMV; *p* = 0.001), normalized WM volume (NWMV; *p* < 0.001), normalized hippocampal volume (NHV; *p* < 0.001), and normalized thalamic volume (NTV; *p* < 0.001) compared to HCs. The hippocampus and thalamus showed increased FC in patients compared to HCs (see Supplementary Table 2).

### Sleep disturbances in MS

Twenty-three MS patients (32 %) were classified as having sleep disturbances (see Table 1). Sleep disturbed patients reported higher levels of subjective cognitive problems (*p* = 0.023) compared to patients with normal sleep.

**Table 1:** Demographics of patient groups

	Normal sleeping MS patients (*n* = 48)	Sleep disturbed MS patients (*n* = 23)	*p*
Age, years	44.55 (8.68)	47.98 (7.14)	0.105
F/M	31/17	16/7	0.678
Educational level^a^	6.00 (5.00–6.00)	6.00 (5.00–6.00)	0.150
RRMS/SPMS	36/11^b^	16/7	0.527
Disease duration, years^a^	10.00 (5.00–14.00)	12.00 (6.00–17.00)	0.360
EDSS^a^	3.50 (3.50–5.00)	4.00 (3.50–4.63)	0.443
HADS-A^a^	5.00 (4.00–7.00)	6.00 (4.00–11.00)	0.075
HADS-D^a^	4.00 (2.00–6.00)	4.00 (3.00–9.00)	0.144
CIS-20	76.63 (28.85)	84.77 (24.72)	0.256
CFS^a^	9.00 (7.00–16.00)	15.00 (12.00–20.00)	0.023

*A* anxiety, *CFS* Cognitive Function Scale, *CIS-20* Checklist of Individual Strength, *D* depression, *EDSS* Expanded Disability Status Scale, *F* female, *HADS* Hospital Anxiety and Depression Scale, *M* male, *RRMS* relapsing-remitting multiple sclerosis, *SPMS* secondary progressive multiple sclerosis

^a^Indicating median and interquartile range instead of mean and SD

^b^ *n * = 47

### Cognition

No differences were found between disturbed and normal sleeping patients with regard to cognitive functioning (see Table 2). Twelve sleep disturbed patients (52 %) were categorized as cognitively impaired versus 14 (29 %) normal sleeping patients (*p* = 0.060).

**Table 2:** Cognitive test scores for normal sleeping and sleep disturbed patients with multiple sclerosis

	Normal sleeping MS patients (*n* = 48)	Sleep disturbed MS patients (*n* = 23)	*p*
Verbal learning and memory			
VLGT—total score	55.50 (44.00–62.75)	52.00 (39.00–59.00)	0.304
Visuospatial memory			
LLT—total number of displacements^a^	16.50 (10.00–30.75)	28.00 (11.00–42.00)	0.169
Information processing speed			
LDST (reading, 90 s)	50.50 (45.00–60.75)	47.00 (38.00–55.00)	0.145
Short term and working memory			
Digit span forward	9.00 (7.00–10.00)^b^	8.00 (7.00–10.75)^c^	0.405
Digit span backward	6.50 (5.00–8.00)	5.00 (4.00–7.00)	0.054
Verbal fluency/memory retrieval			
WLG animals	21.50 (17.25–25.00)	21.00 (19.00–27.00)	0.671
WLG professions	16.00 (13.00–20.00)	16.00 (12.00–19.00)	0.666
WLG m-words	9.00 (6.25–12.00)	9.00 (6.00–10.00)	0.336
Overall *Z*-score	−0.68 (−1.17 to −0.27)	−1.34 (−1.88 to −0.54)	0.073
Cognitively impaired/cognitively preserved	14/34	12/11	0.060

Displayed data are median and interquartile range

*LDST* Letter Digit Substitution Task, *LLT* Location Learning Test, *VLGT* verbal learning and memory task, *WLG* Word List Generation

^a^The higher the score, the worse the performance

^b^ *n* = 46

^c^ *n* = 20

^d^ *χ* ^2^ statistic

### Structural MRI

Disturbed and normal sleeping patients did not differ regarding structural imaging measures (see Table 3).

**Table 3:** Structural magnetic resonance imaging measures for normal sleeping and sleep disturbed patients with multiple sclerosis

	Normal sleeping MS patients (*n* = 48)	Sleep disturbed MS patients (*n* = 23)	*p*
NGMV, L	0.75 (0.05)	0.74 (0.06)	0.806
NWMV, L	0.65 (0.04)	0.67 (0.05)	0.194
T2 Lesion volume, mL	6.39 (6.00)^a^	6.20 (5.40)	0.630
NHV, mL^b^	9.90 (8.29–10.49)	9.62 (9.21–11.23)	0.151
NTV, mL^b^	18.21 (16.59–19.97)	19.49 (16.69–20.58)	0.253

*NGMV* normalized gray matter volume, *NHV* normalized hippocampal volume, *NTV* normalized thalamic volume, *NWMV* normalized white matter volume

^a^ *n* = 44

^b^Indicating median and interquartile range instead of mean and SD

### Functional connectivity

**Fig. 1:**
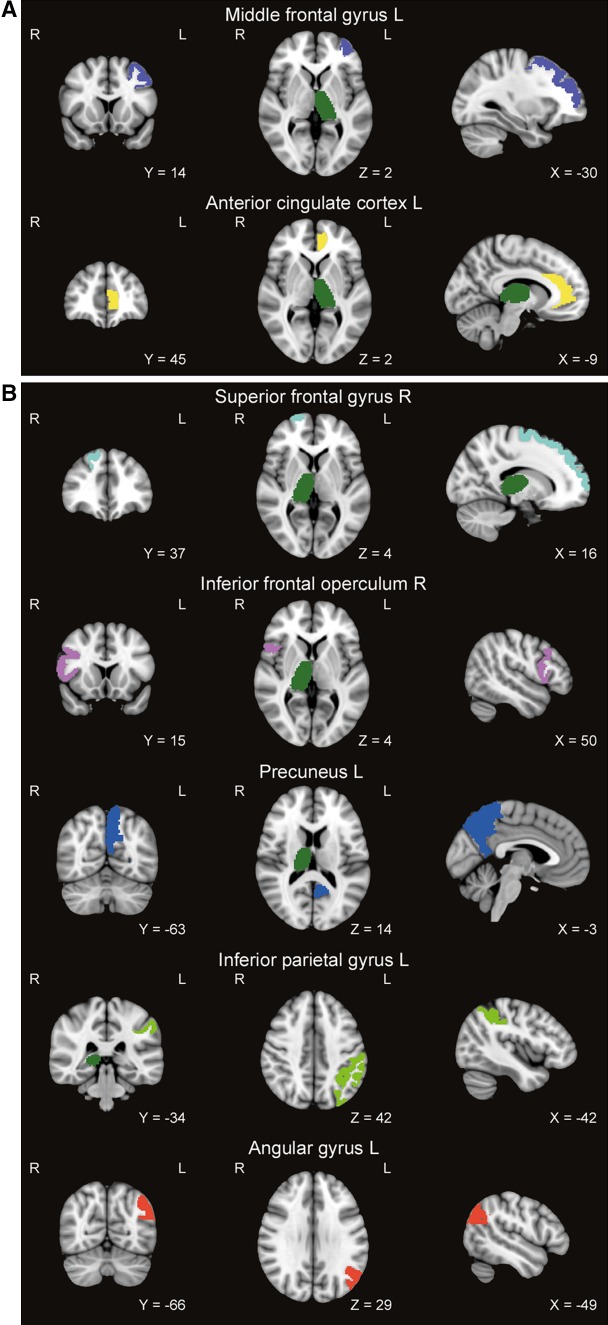
Regions displaying decreased functional connectivity with the thalamus in sleep disturbed compared to normal sleeping patients. For illustrative purposes, the atlas was registered to MNI standard space (1 mm) and brain regions were indicated by different colours. The upper panel (a) displays all connections of the left thalamus that showed decreased functional connectivity in sleep disturbed patients compared to normal sleeping patients. In the lower panel (b), all connections that showed decreased functional connectivity of the right thalamus in sleep disturbed patients compared to normal sleeping patients are visualized *L* left, *R* right

Table 4 displays functional connections that differed between disturbed and normal sleeping patients. Decreased FC was observed in sleep disturbed patients compared to normal sleeping patients between the thalamus and several cortical regions (see Fig. 1). None of the thalamic connections were increased. No differences in hippocampal FC were detected.

**Table 4:** Functional connections that differed between normal sleeping and sleep disturbed patients with multiple sclerosis

	Normal sleeping MS patients (*n* = 48)	Sleep disturbed MS patients (*n* = 23)	*F*	*p*
**Thalamus L**				
Middle frontal gyrus L	0.101 (0.088–0.132)	0.092 (0.073–0.113)	7.203	0.009
Anterior cingulate cortex L	0.102 (0.086–0.127)	0.085 (0.074–0.126)	7.186	0.009
**Thalamus R**				
Superior frontal gyrus R	0.100 (0.083–0.140)	0.083 (0.074–0.101)	8.027	0.006
Inferior frontal operculum R	0.093 (0.083–0.116)	0.081 (0.068–0.097)	7.812	0.007
Precuneus L	0.119 (0.099–0.139)	0.096 (0.083–0.115)	7.714	0.007
Inferior parietal gyrus L	0.101 (0.080–0.127)	0.084 (0.075–0.096)	8.371	0.005
Angular gyrus L	0.080 (0.070–0.096)	0.069 (0.060–0.089)	7.451	0.008

Displayed data are median and interquartile range of untransformed synchronization likelihood

*L* left, *R* right

### Predicting AIS score and cognition in MS

To obtain the *most important * predictors for AIS score in MS, the relationship between those FC measures that differed between disturbed and normal sleeping patients (1/SL), structural brain measures (NGMV, NWMV, NHV, NTV, and T2 lesion load), subjective cognitive problems (confounding variable), and AIS score were assessed using univariate regression analyses. The predictors that survived the univariate regression analyses were: NHV (adj. *R* ^2^ = 0.06, *F* = 5.44, *β* = 0.27, *p* = 0.023), FC between the left thalamus and left anterior cingulate cortex (adj. *R* ^2^ = 0.05, *F* = 4.36, *β* = 0.24, *p* = 0.040), FC between the right thalamus and left inferior parietal gyrus (adj. *R* ^2^ = 0.12, *F* = 9.21, *β* = 0.34, *p* = 0.003), left precuneus (adj. *R* ^2^ = 0.07, *F* = 6.56, *β* = 0.30, *p* = 0.013), left angular gyrus (adj. *R* ^2^ = 0.05, *F* = 4.84, *β* = 0.26, *p* = 0.031), right inferior frontal operculum (adj. *R* ^2^ = 0.13, *F* = 9.91, *β* = 0.35, *p* = 0.002), and right superior frontal gyrus (adj. *R* ^2^ = 0.08, *F* = 7.46, *β* = 0.31, *p* = 0.008). Multivariate backward regression analysis revealed that 16.3 % of variance in AIS score (*F* = 7.83, *p* = 0.001) could be explained by FC (1/SL) between the right thalamus and right inferior frontal operculum (*β* = 0.34, *p* = 0.003) and NHV (*β* = 0.25, *p* = 0.026).

Subsequently, univariate regression analyses were performed to identify the most important predictors for the overall cognitive functioning in MS, including the following variables: age, sex, educational level, subjective cognitive problems, NGMV, NWMV, NHV, NTV, and T2 lesions load. The predictors that survived the univariate regression analyses were: educational level (adj. *R* ^2^ = 0.10, *F* = 8.40, *β* = 0.33, *p* = 0.005), NGMV (adj. *R* ^2^ = 0.07, *F* = 6.46, *β* = 0.29, *p* = 0.013), NWMV (adj. *R* ^2^ = 0.08, *F* = 7.06, *β* = 0.31, *p* = 0.010), NHV (adj. *R* ^2^ = 0.10, *F* = 8.59, *β* = 0.33, *p* = 0.005), and NTV (adj. *R* ^2^ = 0.12, *F* = 10.53, *β* = 0.36, *p* = 0.002). A multivariate backward regression analysis demonstrated that 27.4 % of variance in the overall cognitive functioning (*F* = 9.67, *p* < 0.001) could be explained by the level of education (*β* = 0.42, *p* < 0.001), NHV (*β* = 0.29, *p* = 0.016), and NGMV (*β* = 0.24, *p* = 0.040). Subsequently, AIS score was entered into the model as a second step after the aforementioned predictors. Entering AIS score did not result in an increase in explained variance (*β* = −0.10, *p* = 0.362).

## Discussion

Sleep disturbances and their effects on cognitive functioning and RS FC of the hippocampus and thalamus in MS patients were investigated. In our sample, 32 % of the patients were classified as having sleep disturbances. These patients had similar cognitive profiles compared to normal sleeping patients. Interestingly, decreased FC between the thalamus and the anterior cingulate cortex, precuneus, superior frontal gyrus, middle frontal gyrus, inferior frontal operculum, inferior parietal gyrus, and angular gyrus was found in patients with sleep disturbances compared to normal sleeping MS patients.

Contrary to our hypothesis, we did not observe a difference in objective cognitive functioning between patients with and without sleep disturbances that had an equal level of education and similar structural MRI measures. In our HCs, only information processing speed was negatively correlated with AIS score with a minor remark that our sample had limited variation in AIS score (6/40 HCs were defined as sleep disturbed).

In line with the literature, we found that sleep disturbed patients reported increased subjective cognitive problems. This was previously found in a large sample of 5171 HCs, in which the relationship between sleep disturbances and subjective cognitive functioning was stronger than that with objective cognitive functioning [31].

Although the literature is scarce, it was previously shown that subjective sleep problems in MS patients (with unknown disease duration) could be related to a decline in sustained attention during sequential sessions of a working memory task [9], whereas another study related poor sleep efficiency (measured using polysomnography and a multi-sleep latency test) to worse global cognitive performance (especially executive function and information processing) [5]. In the latter study, 32 patients (mean disease duration 7.5 years) were included and all treated with natalizumab. The difference in patient characteristics might explain the opposite findings since patients in the current study had an average disease duration of 10 years and varied concerning medication. Differences in the sleep measurements (i.e., questionnaires versus polysomnography) and samples might explain the discrepancy in results. Unfortunately, the previous studies did not include MRI to investigate the underlying brain mechanisms of sleep problems.

With regard to FC, patients with MS displayed exclusively *increased FC* in 22 hippocampal and thalamic connections relative to HCs, of which seven were related to the overall cognitive functioning (data not shown). With regard to sleep, patients with sleep disturbances showed *decreased* FC of thalamic connections compared to normal sleeping patients. In addition, the most important predictors for sleep disturbances in MS were: reduced FC between the right thalamus and right frontal operculum and larger hippocampal volume. The latter observation might be explained by the use of a self-report questionnaire to assess sleep disturbances. Although the link between self-report questionnaires and hippocampal volume has not been investigated to our knowledge, a previous study in MS found a similar relationship between larger hippocampal volume and higher levels of self-reported cognitive problems [32].

To our knowledge, this is the first study that investigated sleep disturbances in MS and its association with FC changes. Studies in HCs showed a link between decreased FC and sleep disturbances. For example, after sleep deprivation, decreased FC can be observed in the default mode network [33] and the thalamus [12]. Decreased FC between the thalamus and other regions, such as the superior frontal gyrus, gyrus rectus, precentral gyrus, postcentral gyrus, middle temporal gyrus, and anterior occipital lobe, was also found to be related to daytime sleepiness in an epidemiological study [34]. In the current study, we observed a decrease in FC of the thalamus in sleep disturbed patients, especially in connections between the thalamus and frontal areas (superior and middle frontal gyrus and inferior frontal operculum). This observation is in line with findings in HCs with sleep disturbances.

Thalamic connections that are affected when having sleep disturbances are different from the thalamic connections that are hampered when having cognitive disturbances in MS. In addition, a decrease in connectivity is seen for the sleep disturbed patients, while in relation to cognitive impairment, increased connectivity is mostly reported [14]. Patterns of decreased thalamocortical FC have also been observed in sleep deprived HCs [12]. Although it is not completely elucidated what the underlying mechanism is, in sleep deprived HCs, it was previously suggested to be a result of a decrease in brain metabolism (especially in frontal regions and the thalamus) as measured with positron emission tomography [35], possibly resulting in less synchronized firing of neurons. Our study suggests that a lack of sleep is related to highly specific changes in thalamic-cortico connectivity, which is not directly related to cognitive performance in MS patients.

One possible explanation for the absent relationship between sleep disturbances and cognitive performance might be that patients with severe sleep disturbances were not included in this sample, as subjects were not recruited based on their sleeping behavior. It can be hypothesized that severely sleep disturbed MS patients will be more similar to sleep deprived HCs, and perhaps do show impaired cognition. In our sample, the percentage of patients with sleep disturbances (32 %) was lower than reported previously (~50 %) [6]. The different numbers might be explained by the use of different self-report questionnaires. In the present study, we used the AIS which is a self-report questionnaire that has been validated in HCs [18]. Although it has not been validated in MS, it has been administered in other diseases, such as Alzheimer’s disease [36], which warrants its use in MS. Furthermore, the internal validity of the questionnaire in the present sample (Cronbach’s alpha) for MS patients and HCs was 0.70 and 0.74, respectively. The included items of the AIS assess problems with quality and quantity of sleep, and are not specific for the type of sleep problems that can be found due to MS (e.g., spasticity, sleep apnea, or pain). As no definition has been previously published for the five items version of the AIS, we defined sleep disturbances as scoring at least three points (i.e., median score of patients) with the prerequisite of scoring moderate to severe on at least one item, thereby aiming to be a bit more conservative than using, for instance, a median split approach. A previous study has shown that objective measures of sleep disturbances, such as obstructive sleep apnea, can be related to cognitive dysfunction in MS [37]. Hence, objective measures to quantify sleep disturbances, such as polysomnography, might give a more precise reflection than a self-report questionnaire of the sleep deficits being present. However, we do not expect that if we would have included objective measures of sleep disturbances, patients would have been categorized entirely different.

While FC changes in sleep disturbed MS patients follow a similar pattern compared to changes in connectivity in sleep deprived HCs, it might well be that the effect on cognitive performance is absent due to brain damage caused by MS. Our results suggest that educational level, hippocampal volume, and GM volume can predict overall cognitive functioning. Adding AIS score into the model did not improve the prediction of overall cognitive functioning. Hence, we hypothesize that the severity of structural brain damage in MS patients might be of more influence on cognition than the presence of sleep disturbances. That is, cortical and subcortical GM pathology, but also WM abnormalities, has been linked to impaired cognition in MS. The (widespread) structural brain abnormalities might limit the additional effect of sleep disturbances on cognition. It would be interesting to investigate an early cohort of patients with relatively mild brain pathology to see if sleep disturbances in that stage of the disease do (still) explain part of the cognitive deficits.

In summary, sleep disturbances, as measured with the AIS, in MS do not directly relate to objective cognitive functioning, but rather to subjective cognitive problems. The distinct FC pattern of the thalamus of sleep disturbed MS patients should be investigated in more depth to understand the complex interplay between sleep, cognition, and brain pathology in MS.

## Electronic supplementary material

Below is the link to the electronic supplementary material.

Supplementary material 1 (DOCX 14 kb)

Supplementary material 2 (DOCX 15 kb)

Supplementary material 3 (DOCX 15 kb)
