# Supplementary material for: The role of sleep on cognition and functional connectivity in patients with multiple sclerosis
Source: J Neurol. 2016 Oct 24;264(1):72–80. doi: 10.1007/s00415-016-8318-6 (PMC5225184; doi:10.1007/s00415-016-8318-6)
Supplement: Supplementary file 1 — Supplementary material 1 (DOCX 14 kb) [file 415_2016_8318_MOESM1_ESM.docx]

**ONLINE RESOURCES**

Pre-processing of RS fMRI data was performed using standard FSL protocols, including motion correction, spatial smoothing (5 mm full-width-at-half-maximum Gaussian kernel) and high-pass filtering (100 seconds cut-off). For each subject, an atlas in subject fMRI space was created. Firstly, the Automated Anatomical Labeling atlas (AAL)[1] in standard space was registered to each subject’s pre-processed RS fMRI scan by inverting and applying the boundary-based registration matrix (subject’s fMRI to standard space) that was derived from the data pre-processing steps. Secondly, the atlas was masked with individual GM masks obtained by SienaX. Finally, the subcortical brain regions that were derived from FIRST were registered to the subject’s fMRI space and merged with the AAL atlas, resulting in an individualized atlas containing 92 regions.

Average time series were calculated for each brain region and FC was calculated between the hippocampus and thalamus (bilateral) and all other brain areas using synchronization likelihood (SL)[2] in BrainWave (http://home.kpn.nl/stam7883/index.html). SL is a measure for linear and nonlinear correlations and ranges from zero to one, and has been previously applied in MS[3] and Alzheimer’s disease.[4]

**SUPPLEMENTARY REFERENCES**

1 Tzourio-Mazoyer N, Landeau B, Papathanassiou D, et al. (2002) Automated anatomical labeling of activations in SPM using a macroscopic anatomical parcellation of the MNI MRI single-subject brain. Neuroimage 15:273-289.

2 Stam CJ, van Dijk BW (2002) Synchronization likelihood: an unbiased measure of generalized synchronization in multivariate data sets. Physica D 163:236-251.

3 Hulst HE, Schoonheim MM, Van Geest Q, Uitdehaag BM, Barkhof F, Geurts JJ (2015) Memory impairment in multiple sclerosis: Relevance of hippocampal activation and hippocampal connectivity. Mult Scler 21:1705-1721

4 Sanz-Arigita EJ, Schoonheim MM, Damoiseaux JS, et al. (2010) Loss of 'small-world' networks in Alzheimer's disease: graph analysis of FMRI resting-state functional connectivity. PLoS One 5:1-14.
