# Supplementary material for: The role of sleep on cognition and functional connectivity in patients with multiple sclerosis
Source: J Neurol. 2016 Oct 24;264(1):72–80. doi: 10.1007/s00415-016-8318-6 (PMC5225184; doi:10.1007/s00415-016-8318-6)
Supplement: Supplementary file 2 — Supplementary material 2 (DOCX 15 kb) [file 415_2016_8318_MOESM2_ESM.docx]

**Supplementary Table 1** Demographics and neuropsychological test scores of patients with multiple sclerosis and healthy controls

|  | MS patients (*n* = 71) | Healthy controls (*n* = 40) | *p* |
| --- | --- | --- | --- |
| Age (years) | 45.67 (8.32) | 44.03 (9.14) | 0.338 |
| F/M | 47/24 | 26/14 | 0.898 |
| Educational level^a^ | 6.00 (5.00 – 6.00) | 6.00 (5.00 – 6.00) | 0.627 |
| RRMS/SPMS | 52/18 | – | – |
| Disease duration (years)^a^ | 10.00 (6.00 – 15.25) | – | – |
| EDSS^a^ | 3.50 (3.50 – 4.75) | – | – |
| HADS-A^a^ | 5.00 (4.00 – 8.00) | 3.00 (2.00 – 5.75) | 0.001 |
| HADS-D^a^ | 4.00 (2.00 – 8.00) | 1.00 (0.00 – 2.75) | <0.001 |
| CIS-20^a^ | 71.00 (47.75 – 88.25) | 26.50 (17.50 – 51.25) | <0.001 |
| CFS^a^ | 12.00 (7.00 – 17.00) | 5.00 (3.00 – 8.00)^b^ | <0.001 |
| AIS^a^ | 3.00 (1.00 – 4.00) | 1.00 (0.00 – 1.00) | 0.002 |
| **Verbal learning and memory**  VLGT – total score^a^ | 55.00 (43.00 – 62.00) | 62.00 (55.00- 67.00) | <0.001 |
| **Visuospatial memory**  LLT – total number of displacements^a^ | 21.00 (10.00 – 34.00) | 9.00 (5.25 – 19.50) | <0.001 |
| **Information processing speed**  LDST (reading, 90 seconds)^a^ | 50.00 (43.00 – 59.00) | 63.00 (59.00 – 69.75) | <0.001 |
| **Short term and working memory**  Digit span forward^a^  Digit span backward^a^ | 9.00 (7.00 – 10.00)  6.00 (5.00 – 8.00) | 10.00 (8.00 – 12.00)  8.00 (7.00 – 8.75) | 0.021  <0.001 |
| **Verbal fluency/memory retrieval**  WLG animals^a^  WLG professions^a^  WLG m-words^a^ | 21.00 (18.00 – 25.00)  16.00 (13.00 – 20.00)  9.00 (6.00 – 12.00) | 26.00 (22.00 – 31.00)  19.00 (16.00 – 23.75)  12.00 (9.00 – 14.75) | <0.001  0.006  0.001 |

Abbreviations: A = anxiety; AIS = Athens Insomnia Scale; CFS = Cognitive Function Scale; CIS-20 = Checklist of Individual Strength; D = depression; EDSS = Expanded Disability Status Scale; F = female; HADS = Hospital Anxiety and Depression Scale; LDST = Letter Digit Substitution Task; LLT = Location Learning Test; M = male; RRMS = relapsing-remitting multiple sclerosis; SPMS = secondary progressive multiple sclerosis; VLGT = verbal learning and memory task; WLG = Word List Generation.

^a^ Indicating median and interquartile range instead of mean and SD.

^b^ *n* = 39.
