# Supplementary material for: The role of sleep on cognition and functional connectivity in patients with multiple sclerosis
Source: J Neurol. 2016 Oct 24;264(1):72–80. doi: 10.1007/s00415-016-8318-6 (PMC5225184; doi:10.1007/s00415-016-8318-6)
Supplement: Supplementary file 3 — Supplementary material 3 (DOCX 15 kb) [file 415_2016_8318_MOESM3_ESM.docx]

**Supplementary Table 2** Functional connections that significantly differed between patients with multiple sclerosis and healthy controls

|  | MS patients (*n*=71) | Healthy controls (*n*=40) | *F* | *p* |
| --- | --- | --- | --- | --- |
| **Hippocampus L** |  |  |  |  |
| Gyrus rectus L | 0.085 (0.072 – 0.107) | 0.076 (0.068 – 0.087) | 7.930 | 0.006 |
| Olfactory cortex L | 0.079 (0.070 – 0.100) | 0.069 (0.060 – 0.081) | 9.503 | 0.003 |
| Heschl’s gyrus L | 0.081 (0.068 – 0.103) | 0.070 (0.061 – 0.090) | 7.254 | 0.008 |
| Inferior temporal gyrus L | 0.093 (0.077 – 0.113) | 0.083 (0.068 – 0.095) | 7.197 | 0.008 |
| Middle temporal gyrus L | 0.102 (0.081 – 0.126) | 0.091 (0.074 – 0.110) | 7.070 | 0.009 |
| Posterior cingulate cortex R | 0.090 (0.083 – 0.116) | 0.084 (0.069 – 0.104) | 7.987 | 0.006 |
| Thalamus L | 0.090 (0.081 – 0.117) | 0.085 (0.067 – 0.098) | 10.506 | 0.002 |
| Nucleus accumbens L | 0.079 (0.068 – 0.095) | 0.067 (0.059 – 0.747) | 11.221 | 0.001 |
| Thalamus R | 0.089 (0.078 – 0.115) | 0.079 (0.067 – 0.096) | 7.471 | 0.007 |
| **Hippocampus R** |  |  |  |  |
| Heschl’s gyrus L | 0.086 (0.075 – 0.107) | 0.070 (0.064 – 0.094) | 8.705 | 0.004 |
| Superior frontal orbital R | 0.088 (0.075 – 0.123) | 0.077 (0.065 – 0.094) | 6.883 | <0.01 |
| Inferior frontal orbital R | 0.099 (0.079 – 0.126) | 0.087 (0.071 – 0.104) | 6.888 | <0.01 |
| Heschl’s gyrus R | 0.088 (0.070 – 0.123) | 0.072 (0.061 – 0.093) | 8.270 | 0.005 |
| Middle temporal gyrus R | 0.120 (0.087 – 0.160) | 0.096 (0.082 – 0.120) | 7.663 | 0.007 |
| Nucleus accumbens L | 0.082 (0.071 – 0.100) | 0.070 (0.064 – 0.082) | 12.992 | <0.001 |
| Caudate nucleus R | 0.082 (0.070 – 0.102) | 0.069 (0.063 – 0.087) | 8.308 | 0.005 |
| **Thalamus L** |  |  |  |  |
| Parahippocampal gyrus R | 0.091 (0.078 – 0.121) | 0.079 (0.070 – 0.090) | 8.824 | 0.004 |
| Amygdala L | 0.083 (0.069 – 0.107) | 0.070 (0.059 – 0.079) | 14.359 | <0.001 |
| Nucleus accumbens L | 0.082 (0.069 – 0.093) | 0.071 (0.058 – 0.082) | 11.150 | 0.001 |
| Amygdala R | 0.080 (0.067 – 0.096) | 0.070 (0.061 – 0.081) | 7.861 | 0.006 |
| **Thalamus R** |  |  |  |  |
| Olfactory cortex L | 0.075 (0.067 – 0.089) | 0.070 (0.061 – 0.082) | 7.481 | 0.007 |
| Parahippocampal gyrus R | 0.094 (0.077 – 0.111) | 0.076 (0.064 – 0.093) | 11.652 | 0.001 |

Displayed data are median and interquartile range of untransformed synchronization likelihood.

Abbreviations: L = left; R = right.
